# Energy Recovery from Biowaste and Biomass via Gasification: A Modelling Approach

**DOI:** 10.3390/biotech15010001

**Published:** 2025-12-19

**Authors:** Shabnam Ghanbarzadeh, Yi Yuan, Ehssan H. Koupaie

**Affiliations:** 1 Waste & Wastewater Biorefinery Lab (WWBL), Department of Chemical Engineering, Queen’s University, 19 Division Street, Kingston, ON K7L 2N9, Canada; 23cw53@queensu.ca; 2 Department of Mechanical and Material Engineering, Western University, London, ON N6A 5B9, Canada；

**Keywords:** biowaste gasification, wastewater sludge, food waste, softwood biomass, thermochemical conversion, syngas composition, energy efficiency, aspen plus modeling, circular bioeconomy, waste-to-energy

## Abstract

The transition toward a circular bioeconomy requires efficient conversion of biogenic wastes and biomass into renewable fuels. This study explores the gasification potential of wastewater sludge (WWS) and food waste (FW), representing high moisture-content biowastes, compared with softwood (SW), a lignocellulosic biomass reference. An Aspen Plus equilibrium model incorporating the drying stage was developed to evaluate the performance of air and steam gasification. The effects of temperature (400–1200 °C), equivalence ratio (ER = 0.1–1), and steam-to-biomass ratio (S/B = 0.1–1) on gas composition and energy efficiency (EE) were examined. Increasing temperature enhanced H_2_ and CO generation but reduced CH_4_, resulting in a maximum EE at intermediate temperatures, after which it declined due to the lower heating value of the gases. Although EE followed the order SW > FW > WWS, both biowastes maintained robust efficiencies (60–80%) despite high drying energy requirements. Steam gasification increased H_2_ content up to 53% (WWS), 54% (FW), and 51% (SW) near S/B = 0.5–0.6, while air gasification achieved 23–27% H_2_ and 70–80% EE at ER ≈ 0.1–0.2. The results confirm that wet bio-wastes such as WWS and FW can achieve performance comparable to lignocellulosic biomass, highlighting their suitability as sustainable feedstocks for waste-to-syngas conversion and supporting bioenergy integration into waste management systems.

## 1. Introduction

The growing global energy demand has led to extensive reliance on fossil fuels, which deplete natural resources and contribute significantly to greenhouse gas (GHG) emissions [1]. Petroleum, natural gas, and coal supply approximately 85% of the energy required for the residential and commercial sectors [2]. However, their combustion remains the primary source of CO_2_ emissions, accounting for approximately 70% of total emissions [3]. As concerns over energy security and environmental pollution intensify, the urgent need for sustainable alternatives has become a global priority [4]. Consequently, the transition toward renewable energy sources, which are abundant and highly efficient, has gained significant attention [5].

Wastewater sludge (WWS), a by-product of wastewater treatment plants, contains organic matter, nutrients, and pollutants [6]. Its high levels of phosphorus and nitrogen make conventional treatment methods environmentally harmful [7]. Despite processing billions of tons annually, a large portion of WWS still ends up in landfills [1]. Similarly, food waste (FW) accounts for a significant share of municipal solid waste, and rapid urbanization and population growth are expected to push global MSW generation to 9.5 billion tons by 2050 [8]. This growing waste production poses challenges for both industrialized and developing nations, underscoring the need for sustainable waste management. Organic waste, such as WWS and FW, has the potential for energy recovery and soil fertilization [9]. Traditionally, direct combustion and land application have been used for power generation and soil enrichment [10]. However, these conventional methods are inefficient, generate harmful by-products [11], and have low recycling rates [12], underscoring the importance of landfill reduction and resource recovery.

Energy conversion methods are technologies used to treat biomass and recover energy, addressing both the rising global energy demand and the reduction in waste from various sources [10]. Gasification is a thermochemical process that converts solid carbonaceous feedstocks into a combustible gas mixture suitable for applications such as integrated gasification combined cycle (IGCC) power systems, combustion engines, turbines, and fuel-cell platforms [13,14,15]. This process provides a more efficient alternative to incineration, which generates high levels of NO_x_ and CO_2_, and to pyrolysis, which requires high temperatures and an inert environment while producing tar [16]. Gasification occurs when carbonaceous materials react with limited amounts of gasifying agents, such as air, oxygen, or steam, to yield combustible gases like H_2_, CO, and CH_4_, with the primary reactions including the water-gas shift reaction and methanation [13]. Supplying a sub-stoichiometric oxidant results in a combustible gas mixture dominated by carbon monoxide, methane, and hydrogen, all of which are flammable [15]. Syngas high heating value (HHV) varies with the feedstock and operating conditions; for example, air-blown systems usually produce low-calorific gas (≈4–7 MJ/m^3^), while oxygen-enriched modes can reach 10–18 MJ/m^3^ [15,17].

Both WWS and FW typically have very high moisture contents, which poses a severe energy penalty to their use in thermochemical processes. For example, WWS often arrives with moisture levels exceeding 70–80% (wet basis), necessitating extensive thermal input for drying before gasification can proceed efficiently [18]. Likewise, FW must be dewatered before gasification, and the enthalpy of vaporization of water can become comparable to or exceed the energy released by combustion if the moisture content is too high [19]. Municipalities and waste management agencies frequently cite the high energy demand for preprocessing (drying) as a significant economic and technical barrier to scaling up sludge or food-waste gasification systems [20]. Most experimental and modelling studies have focused on low-moisture lignocellulosic materials, with comparatively fewer works examining wet organic waste. This simplifies reactor design, reduces complexity, and avoids the enormous energy burden of drying. Lyons Cerón et al. (2021) conducted gasification experiments on spruce, alder, and pine wood with moisture contents typically under 10%, reporting syngas compositions at 750–950 °C and an equivalence ratio (ER) of 0.19–0.38 [21]. The review by Mishra et al. (2021) notes that most gasification literature assumes dry biomass (moisture content <  10–15%) in modelling and experiments to avoid heat penalties [22]. In their “Cutting-edge biomass gasification technologies” review, Sher et al. (2025) also emphasize that most bench- and pilot-scale gasification tests use wood residues or agricultural biomass with low moisture to ensure stable operation [23].

Several simulation studies have investigated biomass gasification using Aspen Plus. Mehdi et al. (2023) simulated sludge gasification with 50% moisture and reported that increasing the temperature from 700 to 1300 °C raised H_2_ from 37 to 51 mol% while decreasing CGE from 95% to 82% [1]. Their results also showed strong sensitivity to steam-to-sludge and air-to-sludge ratios, with optimal H_2_ formation at a steam-to-sludge ratio of 0.35 and severe dilution at air-to-sludge ratios above 0.2. Ramzan et al. (2011) simulated hybrid air–steam gasification of food waste, municipal solid waste, and poultry waste [13]. Their analysis demonstrated strong sensitivity to moisture and steam levels: increasing moisture content from 5% to 40% markedly decreased syngas HHV and cold-gas efficiency, whereas steam injection enhanced hydrogen formation at the expense of increased heat demand [13]. Doherty et al. (2009) developed an Aspen Plus model of a circulating fluidized-bed gasifier to evaluate the effects of ER, temperature, air preheating, and steam injection on biomass gasification performance [15]. The authors found optimal operation at ER = 0.35 and 837–874 °C, where H_2_ and CO reached their highest levels, and cold-gas efficiency peaked at 66%. Their sensitivity analysis showed that air preheating increased H_2_ and CO, especially at low ER. Fernandez-Lopez et al. (2017) simulated the gasification of dairy manure in a dual fluidized-bed gasifier using an equilibrium Gibbs-minimization model in Aspen Plus 24. They evaluated temperature (750–950 °C), gasifying agent (steam or CO_2_), and agent-to-biomass ratio (0.1–2), and found that higher temperatures increased H_2_ and CO production. In contrast, CO_2_ as the gasifying agent produced higher LHV syngas (up to 18 MJ/Nm^3^) at low ratios [24]. Although these simulation studies provide valuable insights into biomass gasification, they focus almost entirely on dry or low-moisture feedstocks. These studies rarely evaluate the implications of the drying energy demand on overall gasification efficiency, even though moisture strongly influences system performance.

Building on the gaps noted above, this study aims to compare two high-moisture organic waste—WWS and FW—with a nearly dry lignocellulosic reference (SW) during gasification. An Aspen Plus equilibrium simulation is used to evaluate not only gas composition but also overall process EE, explicitly accounting for drying energy consumption for high-moisture feeds. This research also analyzes the process’s sensitivity to temperature, equivalence ratio (ER), and steam-to-biomass ratio (S/B) under both air and steam gasification modes, to identify optimal operating conditions and quantify trade-offs between hydrogen maximization and EE.

## 2. Materials and Methods

### 2.1. Feedstock Characterization

In this study, three different feedstocks were evaluated: municipal WWS, FW, and SW. The sewage sludge used was primary sludge obtained from the Ravensview Wastewater Treatment Plant (Kingston, ON, Canada). The FW was prepared in the laboratory to reflect the average composition of household FW in North America [25].

Both proximate and ultimate analyses were performed on each feedstock, and the results are presented in Table 1. The proximate analysis determined the moisture content (MC), volatile matter (VM), fixed carbon (FC), and ash content. Moisture content was determined by drying the samples in an oven at 110 °C for 24 h. Volatile matter and fixed carbon were measured by sequentially heating the dried samples in a furnace, first at 550 °C for 20 min to determine volatile matter following Standard Methods [26], and then at 750 °C for 2 h to estimate fixed carbon using the ASTM D3174–12 procedure [27]. The residue remaining after this final step is considered the ash content. Ultimate analysis was conducted at the Ján Veizer Stable Isotope Laboratory in Ottawa using an Elementar Isotope Cube elemental analyzer (Elementar, Germany). The analyzer determined the carbon, hydrogen, nitrogen, and sulfur content (%C, %H, %N, and %S) of the samples. Each powdered sample (2–200 mg) was combusted at high temperature in the presence of oxygen, and the resulting gases were separated and quantified using adsorption traps and a thermal conductivity detector (TCD). Calibration was performed using certified standards and blind samples to ensure accuracy.

The HHV of each feedstock was estimated based on the elemental composition using the empirical correlation (Equation (1)) proposed by Channiwala and Parikh [28], where all input values for C, H, N, S, O, and ash are expressed as mass percentages on a dry basis.

(1) HHV(MJ/kg)=0.3491∗C+1.1783∗H+0.1005∗S−0.1034∗O−0.0151∗N−0.0211∗Ash

### 2.2. Performance Indicators

In this study, specific definitions and performance indicators were employed to evaluate the gasification process. The yield of each gas (expressed as mol%) was determined based on the simulated stream compositions as described in Equation (2). Gas compositions are reported on a wet basis at standard state (25 °C and 1 atm). In air gasification, the product gas may contain N_2_ because the supplied air consists of approximately 79% N_2_ and 21% O_2_. Accordingly, only the main gas components (H_2_, CO, CO_2_, and CH_4_) are presented in the results. To evaluate the energy performance of the system, an EE metric was defined in Equation (5), which compares the chemical energy content of the produced gas (Equation (3)) with the total energy input, including the chemical energy of the feedstock (Equation (4)), the thermal energy required for drying, and the net heat duty of the decomposition and gasification reactors. A positive net heat duty represents energy consumption (endothermic), whereas a negative duty represents energy release (exothermic). All units are assumed to be adiabatic, with no heat loss to the surroundings, and no external heat recovery is considered. The mass flow rates of all process streams and individual components, and the energy requirements of the dryer and reactors, were obtained directly from the simulation results, with energy values reported in units of MJ/hr. HHVs for the combustible gaseous products were taken as 141.5 MJ/kg for H_2_, 55.5 MJ/kg for CH_4_, and 10.1 MJ/kg for CO at standard state (25 °C and 1 atm) [29].

(2) $$Each\;gas\;yield\left(mole\%\right)=yield\;of\;each\;component*100$$

(3) $${HHV}_{produced\;syngas}\left(\frac{MJ}{hr}\right)=\sum_{i}{mass\;flow\;rate}_{i}\left(\frac{kg}{hr}\right)*{HHV}_{i}(\frac{MJ}{kg})$$

(4) $${HHV}_{feedstock}(\frac{MJ}{hr})={mass\;flow\;rate}_{dry\_feedstock}(\frac{kg}{hr})*{HHV}_{feedstock}(\frac{MJ}{kg})$$

(5) $$Energy\;Efficiency\left(\%\right)=\frac{{HHV}_{produced\;syngas}}{\left({HHV}_{feedstock}+{Duty}_{dryer}+{NetDuty}_{reactors}\right)}*100$$

### 2.3. Aspen Plus Model

**Figure 1:**
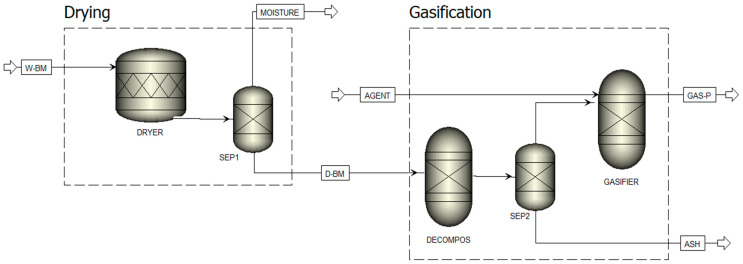
Aspen Plus steady-state flowsheet diagram for biomass conversion to gas via gasification.

A stoichiometric equilibrium model was developed in Aspen Plus V11 to simulate the gasification of three different feedstocks. The process was structured into two main stages, as shown in Figure 1. In the first stage, the non-conventional wet biomass undergoes a drying step to reduce its moisture content. In the second stage, which involves gasification, the dried biomass is first decomposed into its conventional components—volatile matter and ash—of which the latter is separated before entering the reactor. The gasification reactions are then carried out using two gasifying agents, steam and air, and are modelled to minimize Gibbs free energy for gas production.

#### 2.3.1. Model Assumptions

The assumptions in this model are as follows:

- Kinetic-free, steady-state operation;
- Isothermal reactions at atmospheric pressure, no pressure drop;
- No heat loss from the reactor;
- Exclusion of tar formation.

#### 2.3.2. Physical Property Method

The Peng-Robinson equation of state, enhanced with the Boston-Mathias modifications, was used to estimate the physical properties of the conventional components in this model. While the Peng-Robinson method is widely used for hydrocarbon systems, the Boston-Mathias modifications refine the correlation of pure component properties at high temperatures. A key feature of this property package is the temperature-dependent alpha parameter, which significantly improves the accuracy of pure-component vapour-pressure correlations, especially at elevated temperatures [13,30]. The models selected to represent the enthalpy and density of both biomass and ash, treated as non-conventional components, are HCOALGEN and DCOALIGT.

#### 2.3.3. Model Description

The gasification process was modelled using five distinct Aspen Plus blocks (Table 2 and Figure 1). In the simulation, each biomass type was treated as a non-conventional component, characterized based on its ultimate and proximate analyses (Table 1). Initially, the biomass feed enters the system under standard conditions (25 °C, 1 atm) at a mass flow rate of 100 kg/hr. The first stage of the process involves moisture removal, which is simulated using an ‘RStoic’ reactor block to represent the ‘DRYER. ‘The wet feed named ‘W-BM’ is introduced into the dryer at a temperature of 100 °C and a pressure of 1 atm. Drying involves removing moisture from the feedstock, causing some of it to evaporate. To achieve a completely moisture-free and vapour-free solid stream, the feed is subsequently directed into a flash separator named ‘SEP1’. This separator operates at atmospheric pressure and at the same inlet feed temperature, using thermodynamic principles to facilitate flash separation. Before simulating the chemical reactions within the gasifier, the dry biomass, called ‘D-BM’, is broken down into its constituent elements according to its elemental composition (C, H, N, Cl, S, O, Ash) as specified in the ultimate analysis. This decomposition is achieved using the ‘RYield’ block, which performs calculations based on the component yield specification. It is assumed that the total volatile yield corresponds to the volatile content of the original feedstock, as determined by its proximate analysis. The decomposition process occurs at 800 °C and 1 atm pressure. The outlet stream of the ‘RYield’ reactor enters the ‘SEP2’ separator block, which separates gases from ash based on specified split fractions. In the actual gasification process, however, mineral ash is not completely inert and can catalyze key reactions, including R1, R4, R6, and R7. Because the present RGibbs equilibrium model treats ash as a non-reactive solid and therefore removes it before the equilibrium calculations, the catalytic effects of ash are not captured. The stream of decomposed biomass elements is then directed to the ‘RGIBBS’ reactor, known as the ‘GASIFIER. ‘The gasifier operates under the same temperature and pressure conditions as the ‘DECOMPOS’ block, and the heat generated from biomass decomposition is transferred to the ‘RGIBBS’ reactor via a heat stream, simulating the gasification process. An additional ‘AGENT’ stream, serving as the agent gas, is introduced into the reactor, where partial oxidation and gasification reactions outlined in Table 3 occur [17,31]. Certain elements present in the biomass, including chlorine (Cl_2_), nitrogen (N_2_), and sulfur (S), do not participate in the primary gasification reaction equilibrium. To simplify the model, these elements undergo only specific reactions (R8–R10), leading to their complete conversion into hydrogen chloride (HCl), ammonia (NH_3_), and hydrogen sulfide (H_2_S), respectively. It is assumed that all sulfur in the biomass reacts with hydrogen to form H_2_S, although in real systems, small amounts of COS or CS_2_ may also be generated. Given the low sulfur content of the feedstock, any inaccuracies arising from this assumption are considered negligible. Similarly, although nitrogen may form trace amounts of HCN or NOx under practical gasifier conditions, it is assumed here that nitrogen converts exclusively to NH_3_, a simplification commonly adopted in previous studies [13,32]. The gasifier calculates the gas composition stream named ‘GAS-P’ by minimizing Gibbs free energy, assuming the system reaches chemical equilibrium.

### 2.4. Model Validation

To validate the developed simulation model, the operating conditions reported in the literature, including feedstock composition, temperature, gasifying agent type, and the corresponding biomass and agent input amounts, were directly implemented into our Aspen Plus model to reproduce each case. As summarized in Table 4, the predicted gas compositions (H_2_, CO, CO_2_, and CH_4_) obtained under these like-for-like conditions show good agreement with published data, with deviations generally within 4%. The remaining differences arise from inherent variations in model structure and assumptions; for instance, the present model explicitly includes a drying stage and employs equilibrium-based reactors with additional side reactions involving N, Cl, and S (forming NH_3_, HCl, and H_2_S). In contrast, some referenced works relied on experimental setups or simplified simulation schemes. Overall, the close agreement between model predictions and literature values confirms the model’s reliability for further process analysis.

## 3. Results and Discussion

### 3.1. Effect of Gasification Temperature

**Figure 2:**
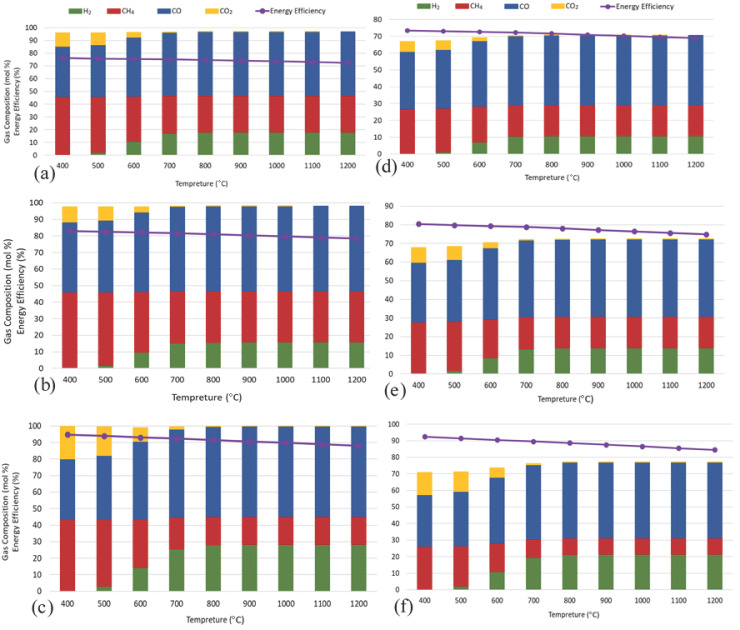
Effect of temperature on gas composition (mol%) and energy efficiency for steam gasification of (**a**) WWS, (**b**) FW, and (**c**) SW with S/B = 0.1, and for air gasification of (**d**) WWS, (**e**) FW, and (**f**) SW with ER = 0.1.

Figure 2 illustrates the effect of temperature on gas composition and EE for the three feedstocks. Panels (a–c) present the results of steam gasification for WWS, FW, and SW, respectively, while panels (d–f) show the corresponding results for air gasification of these feedstocks. Raising the gasification temperature from 400 to 1200 °C increased the total mol% of the major gaseous products, H_2_, CO, CO_2_, and CH_4_, for all feedstocks under both steam and air conditions. This improvement was due to enhanced secondary pyrolysis of volatiles and reactions involved in char gasification, which occur at higher temperatures [37]. At all temperatures, the total mol% of major gases followed the order SW > FW > WWS. This trend closely relates to the VM content of the feedstocks, SW (99 wt.%) > FW (96 wt.%) > WWS (76 wt.%). A higher VM provides more primary volatiles for conversion to permanent gases, whereas low ash content, as in SW and FW, reduces heat transfer and pore-blocking issues that can occur in ash-rich fuels such as WWS (21 wt.% ash). For example, under steam gasification at 800 °C, SW produced about 99.8% of the total mol% of major gaseous compounds, compared to about 98% for FW and 95% for WWS. In a study by Martínez et al. (2014) on the thermodynamic behaviour of gas production from the volatiles of waste tires during pyrolysis, it was observed that raising the gasification temperature from 700 °C to 1200 °C significantly increased the overall gas yield [38]. This enhancement resulted from the promotion of endothermic steam reforming and thermal cracking reactions, which converted a larger portion of condensable hydrocarbons into permanent gases. For example, at 1200 °C, the gas yield was approximately 30% higher than at 700 °C. These modifications also improved the estimated gas heating value, indicating increased proportions of H_2_ and CO in the produced gas [38].

Compositionally, increasing temperature shifted all feeds toward higher CO and H_2_ fractions and lowered CH_4_ and CO_2_ levels in both steam and air gasification because higher heat input accelerated endothermic reactions, such as hydrocarbon gasification (R-1), the Boudouard reaction (R-4), and methane reforming (R-7). Simultaneously, these reactions consumed CH_4_ and CO_2_, causing their levels to decrease as the temperature increased steadily. In steam gasification of FW, H_2_ increased from about 2 vol% at 500 °C to 16 vol% at 800 °C, while CH_4_ dropped from 46 vol% to 30 vol%. Similarly, in air gasification of SW, CO increased from approximately 31 vol% at 400 °C to 41 vol% at 900 °C, whereas CO_2_ decreased from approximately 8 vol% to 0.01 vol%. A similar trend was noted in earlier research. In air–steam gasification of corn straw, the authors concluded that increasing the temperature from 700 to 900 °C raised H_2_ from about 7 vol% to 9 vol% and CO from 15 vol% to 18 vol%. In comparison, CH_4_ and CO_2_ decreased from 6 vol% to 5 vol% and from 17 vol% to 15 vol%, respectively, due to enhanced endothermic reactions [39]. Similarly, in steam–air gasification of sewage sludge, Schmid et al. (2021) reported that higher temperatures increased H_2_ and CO concentrations, accompanied by declines in CH_4_ and CO_2_ [40]. The present results follow the same trend, confirming alignment with these references and other studies in the literature [41].

When comparing gasifying agents, steam gasification consistently yielded higher total mol% of major gaseous outputs and significantly greater H_2_ fractions because steam directly participates in water–gas and steam reforming reactions. In contrast, air gasification produced lower total mol% yields of primary gases and lower H_2_ concentrations due to nitrogen dilution. At the same time, CO levels were often higher at elevated temperatures than in steam gasification because partial oxidation reactions (C + ½O_2_ → CO) became more prominent [42].

The combined effects of feedstock characteristics and gasification pathways can explain the observed EE trends in this study. Across all temperatures and gasifying agents, EE followed the order SW > FW > WWS. The moisture content of the feedstocks primarily influences this ranking, as higher moisture increases the dryer duty in the EE calculation (Equation (4)). WWS, with the highest moisture content, requires significantly more energy for drying, thereby lowering its EE. In contrast, SW’s low moisture content minimizes drying energy demand, resulting in the highest EE values. Given that the HHVs of the feedstocks are relatively similar, temperature effects on EE are mainly driven by changes in the gas HHV. At lower temperatures, EE increases with temperature due to enhanced H_2_ production (notably peaking around 500–700 °C) from water–gas and steam reforming reactions, thereby increasing the gas’s calorific value. Beyond this point, EE declines as CH_4_ and H_2_ concentrations decrease because high-temperature conditions accelerate methane reforming (R-5) and shift the reversible water–gas shift reaction (R-6) toward the reactants, thereby lowering H_2_ levels [43,44].

### 3.2. Effect of Steam-to-Biomass Ratio

**Figure 3:**
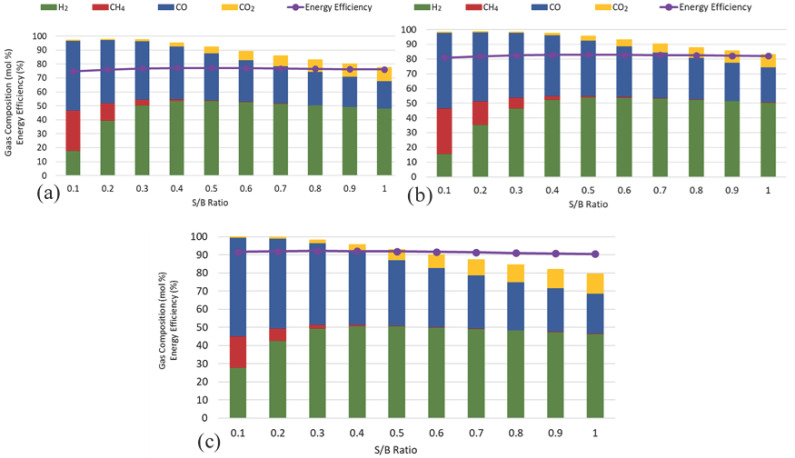
Effect of steam-to-biomass ratio on gas composition (mol%) and energy efficiency for steam gasification of (**a**) WWS, (**b**) FW, and (**c**) SW at a temperature of 800 °C.

The influence of the S/B ratio on the gasification WWS, FW, and SW at 800 °C is presented in Figure 3. The effect of the S/B ratio on gas composition is governed by the interplay between steam reforming and the water–gas shift reaction. Increasing the S/B ratio increased the hydrogen fraction in all feedstocks to an optimal range, while CO and CH_4_ declined steadily and CO_2_ increased moderately. For WWS, the maximum H_2_ concentration of 53% was achieved at an S/B ratio of 0.5; for FW, H_2_ peaked at 54% at 0.6; and for SW, H_2_ reached 51% at 0.5. The EE remained relatively stable across the tested ratios, ranging from 76 to 77% for WWS, 81–83% for FW, and 90–92% for SW, indicating that SW consistently delivered the highest energy efficiency.

This behaviour can be explained by the WGS equilibrium, which shifts toward the production of H_2_ and CO_2_ when additional steam is introduced, and by the steam reforming of methane and light hydrocarbons, which consumes CH_4_ and increases H_2_ yield. The reduction in CO, a significant energy carrier, also explains the observed decrease in gas HHV at higher S/B ratios. Similar results have been widely reported. Gil-Lalaguna et al. (2014) reported that increasing the S/B in sewage sludge gasification enhanced hydrogen content, reaching about 40–45 vol% H_2_ at S/B = 0.6, but simultaneously reduced gas heating value due to significant CO depletion [45]. Yang et al. (2024) numerically studied a dual-fluidized bed. They found a maximum H_2_ yield at a moderate S/B = 0.5, beyond which H_2_ yield and efficiency plateau or decline due to dilution and endothermic load [46]. Consistently, recent thermodynamic-equilibrium modelling (2024) reports that intermediate steam/fuel ratios optimize H_2_/CO via WGS and reforming, while excessive steam flattens HHV, reinforcing the existence of an optimal condition [47]. Collectively, these findings confirm that, under this equilibrium model at 800 °C and the stated boundaries, an optimal S/B ratio of 0.4–0.6 maximizes hydrogen production while balancing enhanced reforming reactions with gas quality and EE.

### 3.3. Effect of Equivalence Ratio

**Figure 4:**
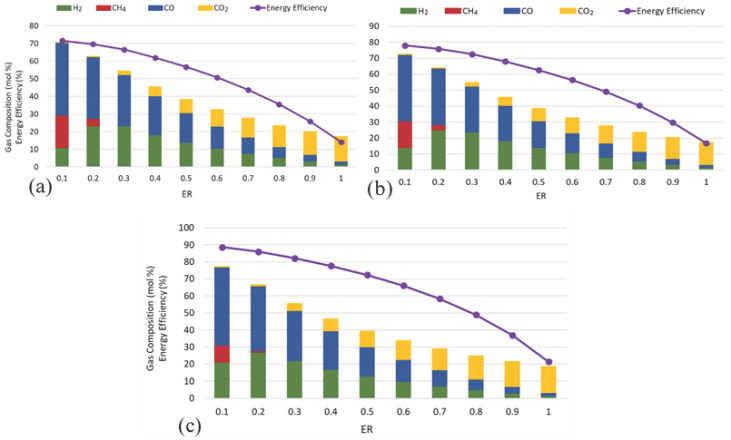
Effect of equivalence ratio on gas composition (mol%) and energy efficiency for air gasification of (**a**) WWS, (**b**) FW, and (**c**) SW at a temperature of 800 °C.

The ER is defined as the ratio of the actual air supplied to the stoichiometric air required for complete combustion of the fuel. In gasification, ER controls the balance between oxidation and reduction reactions and strongly influences gas composition and process efficiency [48]. The influence of ER on the air gasification of WWS (4-a), FW (4-b), and SW (4-c) was investigated at 800 °C. As shown in Figure 4, increasing ER from 0.1 to 0.2 enhanced the H_2_ concentration, with maximum values observed at ER = 0.2 for all feedstocks. Beyond this point, H_2_, along with CO and CH_4_, declined continuously with further increases in ER, while CO_2_ concentration increased progressively across the entire ER range. The highest H_2_ fractions were obtained at ER = 0.2, with values of 23% for WWS, 25% for FW, and 27% for SW. Beyond this point, H_2_ dropped sharply due to over-oxidation. A similar declining trend was observed for EE, which peaked between ER 0.1–0.2 (72% for WWS, 78% for FW, and 88% for SW) and decreased to its lowest values at ER = 1. These results highlight that all feedstocks exhibit optimum gas composition and EE at low ER, with SW consistently yielding the highest efficiency.

These findings are consistent with earlier studies on biomass gasification, where authors reported that increasing ER initially raises the gasification temperature and improves gas production [49,50,51]. Still, excessive oxygen promotes the oxidation of CO and H_2_ to CO_2_ and H_2_O, reducing both H_2_ content and the heating value of the gas. Zhu et al. (2024) demonstrated that higher ER in poplar sawdust gasification decreased the lower heating value (LHV) due to combustion and nitrogen dilution [49], while Susastriawan et al. (2023) found an optimum ER of 0.2 for maximizing HHV and cold gas efficiency in wood-sawdust gasification [50]. Similarly, Saleh et al. (2021) observed that raising ER improved conversion and yield in palm EFB gasification, but LHV declined due to oxidation of combustible gases [51]. These studies confirm that the trends obtained in the present work are typical of air-blown gasification, with optimum performance achieved within a narrow ER window (0.2–0.3) under this equilibrium model at 800 °C and the stated boundaries before over-oxidation and dilution effects dominate

Their proximate and ultimate properties can explain comparative behaviour among the feedstocks. SW contained the highest volatile matter and negligible ash, which facilitated efficient gas-phase reactions and improved gas quality, resulting in the highest H_2_ and EE, as many wood-gasification studies report [52]. FW, despite higher HHV and hydrogen content, exhibited extremely high moisture content, which reduced net energy efficiency but still enabled moderate H_2_ production owing to its high volatile fraction. In contrast, WWS had the lowest HHV and the highest ash content, which diluted combustibles, absorbed heat, and restricted gasification efficiency [53]. These differences explain why SW > FW > WWS in both H_2_ yield and EE.

## 4. Conclusions

This study evaluated the gasification behaviour of WWS and FW as high-moisture-content biowaste streams, compared with softwood (SW) as a dye lignocellulosic benchmark, using an Aspen Plus equilibrium model that incorporated a drying stage and assessed both steam and air gasification modes. The results demonstrated that operating conditions strongly influenced gas composition and energy efficiency across all feedstocks. Increasing temperature enhanced reforming and cracking reactions, increasing H_2_ and improving gas quality, with peak energy efficiencies occurring near 800 °C before declining at higher temperatures due to reduced gas heating value. Despite their high moisture content and associated energy requirements for drying, both FW and WWS maintained competitive energy efficiencies (60–80%) at 800 °C, confirming their potential as viable conversion feedstocks. The influence of steam addition showed a clear optimal range at an S/B ratio of 0.5–0.6, where H_2_ yield ranged from 51 to 54 mol% and energy efficiencies remained high (76–92%). In contrast, air gasification performed best at low ER values (0.1–0.2), yielding 23–27 mol% H_2_ and energy efficiencies of 70–88%, whereas higher ER values led to over-oxidation and nitrogen dilution, reducing gas quality. Across all evaluated conditions, performance consistently followed the order SW > FW > WWS, reflecting differences in volatile matter, ash content, and moisture load. Overall, the findings indicate that optimized operating conditions, centred around 800 °C, S/B = 0.4–0.6, and ER = 0.1–0.2 under the equilibrium model and stated boundaries, maximize H_2_ yield and system energy efficiency. However, differences between simulated and experimental results are expected due to simplified assumptions about carbon conversion and the absence of ash-catalyzed reaction pathways in the model, underscoring the need for refinement to better represent real systems. These results highlight the technical viability of high-moisture wastes for gas production and guide process optimization and scale-up. Future work should extend this framework with techno-economic analysis and system-level integration studies to evaluate the economic competitiveness and sustainability of waste-to-syngas pathways.

## Figures and Tables

**Table 1:** Proximate and ultimate analysis of feedstocks used in this study.

Feedstock	Proximate Analysis (wt.%)							Ultimate Analysis * (wt.%)					Energy (MJ/kg)
	As Received				Dry Basis								
	MC	VM	FC	Ash	VM	FC	Ash	C	H	N	S	O **	
Wastewater Sludge (WWS)	96.1	2.9	0.2	0.8	76.6	2.7	20.8	42.5	5.7	3.8	0.2	27.1	18.3
Food Waste (FW)	94.2	5.6	0.1	0.1	96.5	2.3	1.3	53.1	7.3	2.9	0	35.4	23.4
Softwood (SW)	8.2	91.2	0.6	0	99.3	0.7	0	50.8	6.3	0.2	0	42.8	20.7

* on a dry basis. ** By difference (O% = 100 − (C% + H% + N% + S% + Ash%).

**Table 2:** Description of Aspen Plus units’ operation blocks.

Process ID	Block ID	Temperature (°C)	Description
Dryer	RStoic	100	Reducing the moisture content of the feedstock
Separator 1	Flash2	-	Separating moisture from dry biomass by specifying split fractions
Decomposer	RYield	600	Converting non-conventional biomass into conventional components
Separator 2	Flash2	-	Separating gases from ash by specifying split fractions
Gasifier	RGibbs	600	Calculating gas composition by minimizing Gibbs free energy

**Table 3:** Gasification reactions.

Reaction No.	Reaction Name	Chemical Equation		
R-1	Gasification	C + H_2_O	↔	H_2_ + CO
R-2	Hydrogen combustion	H_2_ + 0.5O_2_	↔	H_2_O
R-3	Carbon combustion	C + O_2_	↔	CO_2_
R-4	Boudouard	C + CO_2_	↔	2CO
R-5	Methanation	C + 2H_2_	↔	CH_4_
R-6	Water-gas shift	CO + H_2_O	↔	CO_2_ + H_2_
R-7	Methane reforming	CH_4_ + H_2_O	↔	CO + 3H_2_
R-8	HCl formation	Cl_2_ + H_2_	↔	2HCl
R-9	NH_3_ formation	0.5N_2_ + 1.5H_2_	↔	NH_3_
R-10	H_2_S formation	S + H_2_	↔	H_2_S

**Table 4:** Model validation by reproducing literature gasification conditions in Aspen Plus.

Source	Feedstock	Temperature	Agent	H_2_(%)	CH_4_(%)	CO_2_(%)	CO(%)
This study	Saw dust	800	Oxygen and Steam	36	0.19	8.8	42
Gasification of Karanja press seed cake [33]				39	0.5	5.2	45
Difference *				−3	−0.31	3.6	−3
This study	Wood residue	700	Steam	45.8	7	8.8	23
Steam gasification of biomass [34]				45	10.5	13.4	25
Difference				0.8	−3.5	−4.6	−2
This study	Groundnut shell	800	Air	60	1.8	1.2	32
Air gasification of groundnut shells [35]				63	2	4	32
Difference				−3	−0.2	−2.8	0
This study	Sewage sludge	1000	Steam	48.7	7.4	6	38
Steam gasification of sewage sludge [36]				45	7	10	35
Difference				3.7	0.4	−4	3

* Absolute difference = model value − literature value.

## Data Availability

The original contributions presented in this study are included in the article. Further inquiries can be directed to the corresponding author.
